# Analysis of clinical, single cell, and spatial data from the Human Tumor Atlas Network (HTAN) with massively distributed cloud-based queries

**DOI:** 10.21203/rs.3.rs-7769205/v1

**Published:** 2025-11-17

**Authors:** David L Gibbs, Dar’ya Pozhidayeva, Yamina Katariya, Boris Aguilar, Kristen Anton, Clarisse Lau, William JR Longabaugh, Ino de Bruijn, Alex Lash, Milen Nikolov, Jennifer Altreuter, Ashley Clayton, Aditi Gopalan, Adam J Taylor, Nikolaus Schultz, Ethan Cerami, Vesteinn Thorsson

**Affiliations:** 1. Institute for Systems Biology, Seattle, WA, USA.; 2. Dartmouth Health, Lebanon, NH, USA.; 3. Memorial Sloan Kettering Cancer Center, New York, NY, USA.; 4. Dana-Farber Cancer Institute, Boston, MA, USA.; 5. Sage Bionetworks, Seattle, W, USA.

## Abstract

Cancer research increasingly relies on large-scale, multimodal datasets that capture the complexity of tumor ecosystems across diverse patients, cancer types, and disease stages. The Human Tumor Atlas Network (HTAN) generates such data, including single-cell transcriptomics, proteomics, and multiplexed imaging. However, the volume and heterogeneity of the data present challenges for researchers seeking to integrate, explore, and analyze these datasets at scale. To this end, HTAN developed a cloud-based infrastructure that transforms clinical and assay metadata into aggregate Google BigQuery tables, hosted through the Institute for Systems Biology Cancer Gateway in the Cloud (ISB-CGC). This infrastructure introduces two key innovations: (1) a provenance-based HTAN ID table that simplifies cohort construction and cross-assay integration, and (2) the novel adaptation of BigQuery’s geospatial functions for use in spatial biology, enabling neighborhood and correlation analysis of tumor microenvironments. We demonstrate these capabilities through R and Python notebooks that highlight use cases such as identifying precancer and organ-specific sample cohorts, integrating multimodal datasets, and analyzing single-cell and spatial data. By lowering technical and computational barriers, this infrastructure provides a cost-effective and intuitive entry point for researchers, highlighting the potential of cloud-based platforms to accelerate cancer discoveries.

## 1. Introduction

As part of the National Cancer Institute (NCI) Cancer Research Data Commons (CRDC), the Institute for Systems Biology Cancer Gateway in the Cloud (ISB-CGC) has developed and made available a substantial collection of data through BigQuery tables^1–3^. Google BigQuery is a columnar database designed to handle extremely large queries and is widely used by large-scale technology companies^4,5^. Its success stems from (1) comparatively low data storage and access costs and (2) massively parallel data processing made possible through ‘Dremel’ technology^6^. This massively parallel architecture enables interactive querying of big data and allows users to develop and refine complex queries iteratively.

Within the Human Tumor Atlas Network (HTAN), data-generating centers are coordinated by a central Data Coordinating Center (DCC), which establishes standards for data annotation and provides infrastructure for both the contribution and dissemination of data to the research community. Specifically, the DCC uses Synapse, a file-based storage system developed and maintained by Sage Bionetworks^7^. In cooperation with the ISB-CGC, the DCC also disseminates diverse assay data as publicly accessible, cloud-based BigQuery tables. A wide variety of data types are available, including phenotypic, sequencing and imaging modalities in single-cell or spatial formats.

In addition to BigQuery tables, we provide a collection of R and Python notebooks demonstrating how to access and analyze HTAN BigQuery data. These notebooks are made publicly available as part of the ISB-CGC Community Notebooks repository. Prior work in the ISB-CGC has shown that summary statistics and various analyses can be efficiently computed on extremely large tables within the Google BigQuery environment. The notebooks leverage both statistical and numerical functions built into BigQuery SQL and user-defined JavaScript functions. These examples include statistical methods such as t-tests and Spearman correlations.

We have also developed novel applications of geospatial analysis for spatial biology data. Using the Earth-based coordinate system, our methods enable a multitude of analyses, such as computing regional summaries or distances between regions or points. By adapting BigQuery’s geospatial functions, we support the analysis and summarization of spatial data, a hallmark of the HTAN project. This approach is applicable to spatial transcriptomics, proteomics, and imaging data generated by protocols such as multiplexed immunohistochemistry, CODEX, MIBI, Visium, and others, resulting in a powerful system operable from any internet-connected device.

## 2. Methods

### 2.1. Transforming data from HTAN

As part of the data submission process, data contributing centers (HTAN Centers) submit assay data files, molecular and cellular measurements, together with metadata detailing assay parameters, the assayed samples, and comprehensive patient clinical information. Metadata is submitted by HTAN Centers as tabular files, where one file is submitted per patient cohort, batch of samples, or collection of assay files.

The processed assay data that are most readily and useful for *most* biological and biomedical analysis are often tabular, and are submitted to the DCC as comma or tab-delimited files. Other submitted assay files, such as sequence read files or imaging data, are either not inherently tabular or of limited utility if converted to tabular form without further processing.

Following each major HTAN data release, the DCC transforms tabular assay and metadata into Google BigQuery tables, hosted by the ISB-CGC under the isb-cgc-bq, part of the “Google Cloud Public Dataset Program” (https://cloud.google.com/bigquery/public-data/). Newly released HTAN data are audited and curated for inclusion in the BigQuery collection. The audit process identifies high-level processed tabular data (e.g., CSV, TSV, H5AD, RDS), excluding files already available in ISB-CGC. Eligible files undergo content analysis to determine their optimal BigQuery table structure. Factors considered include table size, number, and sparsity. Table column descriptions (required by ISB-CGC) are constructed with manual curation from publications when not directly available through the DCC.

When possible, metadata (multiple files from multiple data contributors) is consolidated into a single BigQuery table to enable comprehensive queries. By collecting and combining datasets, we limit the number of table joins when performing a query, making analysis more straightforward. This includes unified tables for all HTAN across all patients (e.g., aggregated demographics data), all samples (biospecimens), and, where possible, all assay data from a particular study (e.g., aggregated gene expression data from single-cell experiments). After each data release, notebooks illustrating use cases are authored and submitted to the ISB-CGC Community Notebooks repository.

Similar to the approach used by The Cancer Genome Atlas (TCGA) consortium^8^, HTAN data are organized into levels based on the degree of processing: *Level 1* contains raw data, *Level 2* includes aligned primary data, *Level 3* consists of derived biomolecular data generated through downstream analysis, and *Level 4* contains sample-level summary data (Figure 2). More information on HTAN data structures can be found in the “The HTAN Manual” (https://docs.humantumoratlas.org/data_model/overview/).

In HTAN, clinical data, sample biospecimen data, and even assay files themselves all have a rich set of metadata annotations supplied by HTAN data contributors. The structure of the annotations aligns with the HTAN Data Model, a set of standards defined by the HTAN consortium.

In the HTAN Phase 1 Data Model, Tier 1 Clinical Data consists of seven main components: demographics, diagnosis, exposure, family history, follow-up, molecular test, and therapy. For example, demographic data across all HTAN Centers is aggregated into a single BigQuery clinical tier 1 demographics table. The same is true for the clinical tier 1 diagnosis table, where each row represents a participant for whom demographic information was reported. In some cases, participant demographic information will appear more than once, indicating a clinical update such as a change in **Vital Status**. As of HTAN Data Release 6.0, there are a total of 2,464 demographic records representing 2,464 distinct participants. See Table 2 for counts of samples per HTAN contributing center.

Prescribed therapies are recorded in the clinical tier 1 therapy table and contain variables such as **Treatment_Type, Treatment_Dose, Treatment_Frequency, and Number_of_Cycles**, among others. This information is available for a subset of patients. As of HTAN Data Release 6, the table contains 1,434 therapy annotations for 535 patients.

### 2.2. BigQuery table tidy structures

BigQuery is a columnar database, containing a set of variables in named columns with measured observations in each row. For high-dimensional data like gene expression, a traditional ‘wide’ format, which would produce thousands of columns, is not practical. Instead, the data is transformed into a ‘long’ or ‘tidy’ format where each row contains a single observation. Using gene expression from single-cell RNA sequencing as an example, each row would contain one gene, one cell barcode, and an expression value. To retrieve all expression values for a given cell, the query traverses the table and selects rows where the cell barcode matches a given search-string (see Supplemental Query examples 1). This long-format table structure enables efficient querying and leverages BigQuery’s extremely powerful columnar architecture, which supports massively parallelized data access and processing capabilities.

### 2.3. Getting started with BigQuery

BigQuery is a Google product and requires a Google Cloud account to access. Fortunately, BigQuery offers a free usage tier. Generally, BigQuery charges are based on the amount of data processed, specifically the number of bytes read per column. Due to the columnar structure, reading fewer columns and rows can directly lower costs. In practice, many users operate well within the free tier, which includes up to one tebibyte (approximately one terabyte) of data per month.

A convenient way to interact with BigQuery tables is through Google *Collaboratory* or *Colab*, an online Python notebook environment. Colab provides free compute resources and simplifies the authentication process, making it a highly accessible option for users. However, local authorization is supported through the pandas-gbq library. The HTAN BigQuery repository contains example notebooks that demonstrate both approaches (Table 1, notebook 1).

### 2.4. R and Python notebooks

As part of the ISB-CGC Community Notebook repository, HTAN provides a collection of R and Python notebooks designed to facilitate the exploration and analysis of clinical, biospecimen, assay, molecular, and spatial data using Google BigQuery. These notebooks guide users in querying and visualizing metadata, identifying data files by organ or cancer type, and analyzing precancer cases. For molecular and cellular data, notebooks demonstrate querying single-cell RNA sequencing data, constructing Scanpy AnnData objects, and analyzing multiplexed imaging and spatial data with BigQuery’s geospatial analytics. Workflow-oriented notebooks also illustrate integrating open-access HTAN data with controlled-access data in CRDC Data Studio using the HTAN ID provenance table. Together, these resources support users in leveraging HTAN data for research and discovery.

## 3. Results

### 3.1. Using the data provenance table for cohort building

The HTAN data generated by HTAN Centers is highly heterogeneous, uses a wide array of technologies, involves multiple stages of processing, and is stored in many different data formats. As a result, preparing for analysis can appear daunting. To help users navigate this landscape, “A Guide to HTAN Data” (Table 1, Notebook 3) provides an introduction to the data ecosystem and demonstrates how to get started with analysis.

The most central item in the collection is the HTAN ID provenance table, a Rosetta Stone that connects the variety of identifiers and names across datasets. This table enables users to query biospecimen and assay files associated with specific patients, tumor types, or cohorts. It also supports the derivation of relationships across different datasets, such as linking single-cell sequencing data to imaging data and patient demographics. The “HTAN ID Provenance in BQ” notebook (Table 1, Notebook 4) introduces the HTAN ID provenance table, demonstrates how it can be accessed, and provides examples of how to use HTAN IDs to link assay data with upstream biospecimen and participant metadata.

### 3.2. Identifying precancer samples and organ-specific data

#### 3.2.1 Organ-specific cohort creation and data discovery

With 10,626 unique biospecimens from 2,083 participants, identifying appropriate samples can prove difficult. To help in this task, we have authored two notebooks that illustrate sample identification (see Table 1, Notebooks 6 & 7). The HTAN data portal is also a valuable resource for identifying cohorts, samples, and associated files through the provided dropdowns and search (https://humantumoratlas.org/explore).

BigQuery tables enhance those capabilities by supporting flexible and unconstrained queries across all variables and values. For example, the clinical tier 1 diagnosis table includes the **Tissue or Organ of Origin** attribute with 332 possible *values*, encompassing both whole organs and organ components. For convenience, a mapping of these 332 **Tissue or Organ of Origin** values to 30 broader organ categories is provided in our BigQuery resource tissue and organ mapping table. To determine what types of assay data are available for a selected anatomical site, several BigQuery tables must be joined, most critically the HTAN ID provenance table and the tissue and organ mapping table. For example, to identify available samples with both single-cell and imaging assay data for breast cancer, one can extract HTAN sample IDs and assay data using a series of SQL joins across key BigQuery tables. This process produces a comprehensive results table that enables users to identify relevant samples and access data in ISB-CGC (Table 1, Notebook 6).

Starting with the anatomical site *breast*, we collect tissues and organs corresponding to *breast* in order to get the 9 representative tissue names. Using this set of tissue names, we query the clinical tier 1 diagnosis table to identify participants who have diagnoses originating in the breast. This query returns 999 HTAN participant IDs. With the list of IDs, we query the HTAN ID provenance table for available assays. The results provide the HTAN data generating center file names and Synapse IDs that can be used to download the data using the CRDC General Commons (GC), the Synapse web portal, the Gen3 client, or using the Synapse command line tool^1,7,9^. In this example, the query identifies 1,333 Level 4 imaging data files and 93 Level 4 single-cell RNA-seq files released for breast cancer patients associated with breast cancer cases released on the HTAN data portal.

#### 3.2.2 Precancer cohort creation and data discovery

Among the 10 HTAN Centers in Phase 1 of HTAN, 5 are designated Precancer Atlases (PCAs)^10^. These centers focus on understanding the molecular and cellular transition phases in pre-malignancy. To identify samples related to precancer studies, we leverage the HTAN Google BigQuery clinical and biospecimen tables to extract relevant cases and specimens (see Table 1, Notebook 7).

Using the HTAN data model, several approaches can be used to identify both patients diagnosed with precancerous lesions and biospecimens collected at the precancer stage of disease. Due to the structure of the data elements pertaining to precancer and the previously set requirements for this data, no single field provides a comprehensive identifier. Instead, identification is achieved through a union of queries across the clinical tier 1 BigQuery tables.

For precancer cases, **Age at Diagnosis**, a required field in the HTAN data model, was set to *θ* to indicate the absence of a cancer diagnosis, providing a specific value for querying. In contrast, the data element **Precancer Condition Type** was not a required field in the data model and thus is not fully annotated for all samples. Atlases contributing data on precancers populate **Age at Diagnosis** with *θ* since participants do not receive a cancer diagnosis. Applying this criterion and focusing on precancer-designated centers, we identified 100 precancer cases from Boston University (BU) and 156 precancer cases from Vanderbilt HTAN centers, all with the primary diagnosis marked as *Not Reported*.

**Primary Diagnosis** is another required clinical data element in the HTAN data model that uses the World Health Organization’s (WHO) International Classification of Diseases for Oncology (ICD-O) to describe the patient’s histologic diagnosis. A limited set of permissible values describes precancers, for example, *Ductal Carcinoma in situ*. Utilizing this data element resulted in an additional 777 recovered cases: 767 cases with disease identified as *Ductal Carcinoma in situ* (Duke University) and 10 cases with disease identified as *Familial Adenomatous Polyposis* (FAP, Stanford University).

To help identify precancerous biospecimens, the required data element **Tumor Tissue Type** can be queried using several permissible values that indicate a precancerous lesion. These include *Premalignant*, *Atypia - hyperplasia*, and *Premalignant - in situ*. Using these values, we identified an additional 1073 precancer biospecimens: 672 *Premalignant*, 248 *Atypia - hyperplasia*, and 153 *Premalignant in situ*. However, some ambiguity remains due to the presence of less specific permissible values, such as *Not Otherwise Specified* (1784 records) and *None* (169 records).

Another approach for identifying samples uses the histologic morphology codes, which are based on the International Classification of Diseases for Oncology, Third Edition (IDC-O-3) coding. These codes offer insight into the classification of the biospecimen. By using this feature, we scan **Histologic Morphology Code** values for premalignancies and associated histologic morphology codes. While most entries for this data element are valid morphology codes, as intended, some are the actual IDC-O-3 codes. However, as data is entered by different organizations, some inconsistencies across centers are to be expected. After some data scrubbing, we find codes for tubular adenoma (M82110), serrated adenoma (M82130), tubulovillous adenoma (M82630), and the benign condition of adenomatous polyposis coli (8220/0). No other codes indicating premalignancy have been reported to date, but this nets an additional 107 samples.

Using the queries outlined above, we identified a set of 2,029 HTAN precancerous cases. While this set may not be exhaustive due to current data limitations, it reflects the available cases and specimens annotated as precancerous across participating centers. The complete list of HTAN IDs, along with the exact queries used, can be found in Table 1, Notebook 7. The HTAN DCC is actively refining the data model to facilitate more direct and comprehensive querying of precancer cases and specimens for HTAN Phase 2.

### 3.3. Multi-modal cohort construction and discovery

Multiple assay types are often available for a given HTAN sample, providing the opportunity for cross-modal analysis. For example, training AI vision models requires pairing imaging data with another assay or clinical data as machine learning targets. To demonstrate multi-modal data discovery and cohort building, we provide a notebook illustrating sample identification (see Table 1, Notebooks 8). It includes two examples: one linking imaging data with spatial assays, and another linking imaging data with sequencing assays.

Image data is linked to cloud storage using **DRS_URI**, which follows the GA4GH Data Repository Service (DRS) specification. These standardized identifiers offer a consistent way to reference and access data files stored across cloud-based research commons such as the CRDC.

The **DRS_URI** (e.g., drs://dg.4DFC:object-id) provides a standardized way to reference data across cloud platforms, allowing tools like Gen3 or Terra to locate and download data without revealing its physical storage location. This enables authorized users to integrate controlled imaging data into cloud-based workflows while ensuring compliance with data protection policies.

In the first example, we build a cohort assayed with both H&E and MxIF imaging techniques. This is achieved by matching metadata from two imaging types using the biospecimen ID as a common linkage key. Selecting records from the imaging level 2 table that have a non-null **Data_Release** and an **Imaging_Assay_Type** of either *H&E* or *MxIF*, grouping by **HTAN_Center** and **HTAN_Parent_Biospecimen_ID**, gives us a table that we can filter, keeping only those biospecimens that have both *H&E* and *MxIF* assay types. This table can then be re-joined to the original imaging level 2 table to retrieve the associated file information, linking with our **DRS_URI** mapping table.

In the second example, we match an imaging modality to another non-imaging assay type. Specifically, we use H&E images and sequencing data from the scRNA-seq level 3 table. Unlike the imaging metadata, the scRNA-seq level 3 table does not include the **HTAN_Biospecimen_ID**, requiring the use of the HTAN ID provenance table (see section 3.1). By joining the provenance table with the sequencing metadata, we retrieve the corresponding **Biospecimen ID** for each record. These can then be joined to an H&E imaging metadata table to identify matched samples. The result is then merged with the **DRS_URI** mapping table to obtain file access links.

Finally, in order to demonstrate the process of accessing and downloading the data, we provide a notebook generating a download manifest that can be used with the CRDC General Commons / Cancer Genomics Cloud Powered by Seven Bridges (CGC), see Table 1, Notebooks 9.

### 3.4. Single-cell data analysis using BigQuery

The HTAN BigQuery resource also includes both single-cell transcriptomics (scRNA-seq) and epigenomics (scATAC-seq) in patient merged tables. In these tidy formatted tables, each row corresponds to counts for a single gene (for scRNA-seq) or genomic location (for scATAC-seq) in a single patient from a single center (see Table 1, Notebooks 10–13).

With direct access to gene expression counts via BigQuery SQL, we can flexibly query data and construct Scanpy AnnData^11^ or Seurat^12^ objects. We can use these particular packages to produce clusterings or perform differential analysis. A complete demonstration of this process is provided in Table 1, Notebook 10.

By building standardized data structures with the AnnData library, we can apply packages such as CellTypist, an open-source tool for automated cell type annotations^13^. In Notebook 12, HTAN single-cell RNA sequencing data (MSK level 4 scRNA-seq, gene summarized, MSK HTAN Center) was accessed via BigQuery. This dataset contains 155,098 transcriptomes from 21 human biospecimens, including 54,523 small cell lung cancer (SCLC) transcriptomes^14^. Using this data, a single-cell atlas of SCLC patient tumors was constructed with comparative lung adenocarcinoma (LUAD) and normal lung. A subset of the combined SCLC tumors dataset was selected based on cluster-defined PTPRC (CD45) positivity. This subset contains both raw gene expression counts and log2(X+1) transformed, median-normalized expression values for 16,098 cells.

After constructing the AnnData object, we confirmed that quality control (QC) filtering has already been performed, such as removing cells with low gene counts. Next, we transformed the data as required by CellTypist, performing log1p normalization and scaling counts to 10,000 per cell. Since the dataset contains only immune cells, we selected the low-resolution immune cell model in CellTypist. This model was built from immune sub-populations derived from 20 tissues across 18 studies.

Using default parameters, the predicted CellTypist labels closely align with those reported by the original authors and exhibit high confidence scores (see right-most plot). Notably, cells originally annotated as *Natural Killer* (NK) were predicted as *Innate Lymphoid Cells* (ILCs) by the CellTypist model. Biologically, this makes sense as ILC cells are a supercategory of NK cells^15^. Given that the model used was for a low-resolution immune cell classifier, it is consistent that the NK cells were folded into the ILC label.

With immediate access to single-cell data in BigQuery, the range of analysis possible increases, whether by summarizing data directly with BigQuery using SQL or by building downstream analysis objects like AnnData.

### 3.4. Spatial data analysis.

#### 3.4.1. BigQuery spatial data table formats.

Along with single-cell data from dissociated cells, HTAN provides BigQuery tables containing information on cells in the spatial context (Table 1, Notebooks 14–16). Spatial data in HTAN spans a wide range of assay types and processing stages, from raw imaging (Level 1) to fully processed, summarized data (Level 4). For example, Level 3 spatial data can result from cell segmentations that provide the pixel mask for each cell. Level 4 imaging data typically takes the form of object-by-feature tables, most often cell-by-marker matrices, generated from the segmentation mask and corresponding image. A multitude of imaging platforms yield summarized data in this form.

As an example, Notebook 14 illustrates the analysis of Multiplexed Ion Beam Imaging (MIBI) data^16^ on breast ductal carcinoma in situ (DCIS) from the Duke University HTAN Center (Duke level 4 MIBI imaging), including the recreation of published plots from the published manuscript. The notebook walks through basic image analysis, such as computing cell centroids, as well as essential data transformation steps like table querying, filtering, pivoting, and merging, all focused on **HTAN_Biospecimen_ID** *HTA6_1045_1*. In addition to reproducing figures, the notebook extends analysis by visualizing additional features such as clustering results and demonstrating the utility of the database.

#### 3.4.2. Exploring spatial cellular relationships

**Figure 1:**
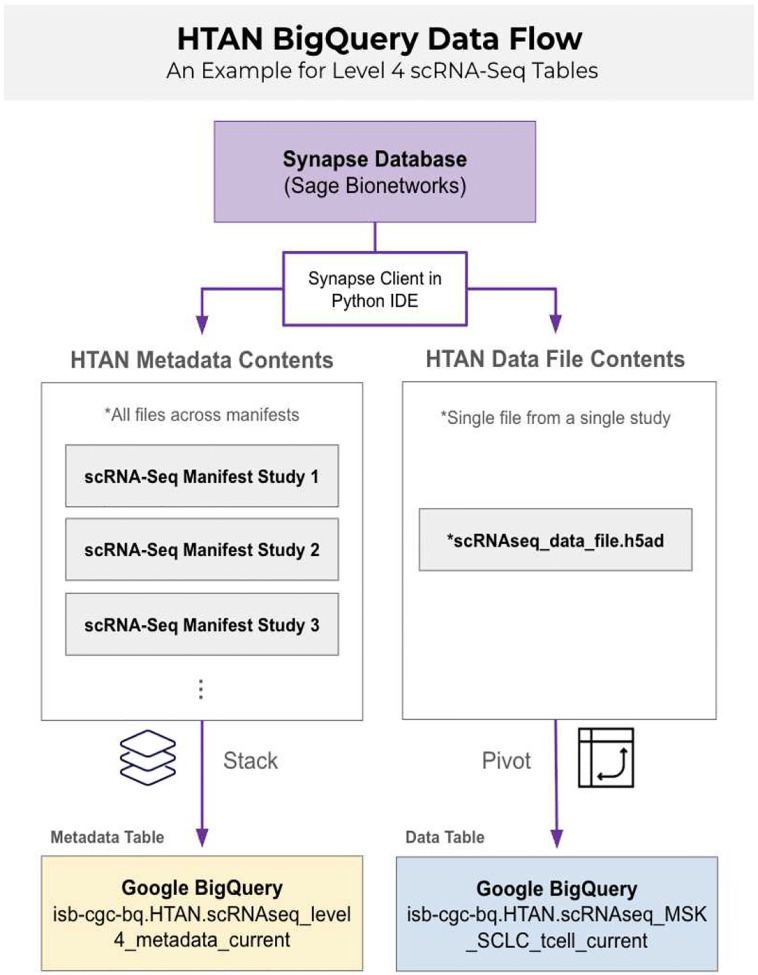
Tabular information in HTAN captured in BigQuery HTAN BigQuery houses two types of data in tabular formats: measurement data (such as transcript counts) and metadata tables. The yellow box represents metadata that has been compiled into a single table for a given level and component (example scRNA-seq Level 4). The blue box represents gene counts from a single count file uploaded to synapse by a data contributor, transformed into a dataframe and uploaded to Google BigQuery.

Beyond data visualization, HTAN spatial BigQuery tables are also useful for computing a range of spatial statistics and metrics. In the following example (Table 1, Notebook 15), we focus on an HTAN colorectal cancer (CRC) biospecimen, *HTA13_1_101*, part of a study investigating dynamic and spatial variation in cancer progression and the tumor microenvironment in CRC^17^ (see e.g. manuscript Figure 1C). The data for this and other CRC tissue sections are available in the HMS level 4 CRC mask table, which contains estimated marker intensities extracted from cell segmentations using three kinds of mask geometries. Here we use the values associated with the **cellRingMask**, defined as a fixed number of pixels around the nuclear segmentation. Cell locations are designated by **X_centroid** and **Y_centroid** and segmented cell geometries are also provided in the table.

The notebook provides examples of how to identify and plot tumor and immune cells in the spatial context. Tumor cells are identified by examining Keratin marker values (**Keratin_570_cellRingMask**) and thresholding the resulting intensity distribution. For illustration purposes, we used the third quartile as the threshold. Using this simple approach, the resulting tumor regions closely resemble those shown in the manuscript. Similarly, we identify immune cells as CD45-positive by thresholding for intensity in the upper quartile. The resulting distribution of immune and tumor cells is observed to be spatially co-located. Tumor cells that have a predominance of neighboring immune cells may represent distinct tumor-immune phenotypes, which could carry biological significance.

Spatial neighborhood analysis provides insight into how different tissue regions compare in cellular content, function, and interactions. Neighborhoods can be defined geometrically, for example, by including cells within a specified radial distance from the center of a reference cell, or by identifying a fixed number of nearest neighbors. As an example, we constructed neighborhoods using 10 nearest neighbors for each cell. To limit resource-intensive pairwise distance calculations, we limit the illustration to a 2500 × 2500 pixel subregion (approximately 1.625 millimeters each side). After querying, subsetting, and downloading the relevant spatial data, analysis can proceed locally. The notebook demonstrates how to obtain the nearest k neighbors of each cell using a kd-tree via the “dbscan” R package. For a particular cell, **CellID** *995338* in this example, the IDs and distance of nearest neighbors, in pixels, can be found through indexing this object.

**Figure 2.**
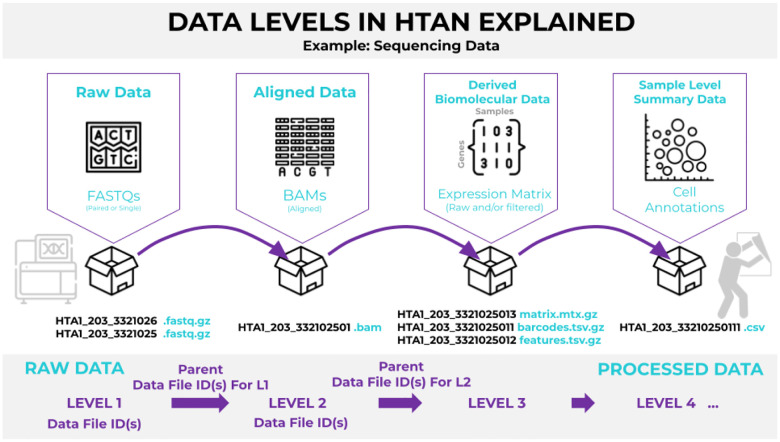
Assay Data Levels in HTAN The assay data in HTAN is divided into distinct levels based on their respective processing pipelines. ISB-CGC BigQuery only houses processed data sets such as Level 3 and Level 4 sequencing.

The original manuscript describes a method for calculating spatial correlation functions between pairs of cell markers, enabling the estimation of the spatial extent of cancer-associated cellular structures. This correlation, examined as a function of distance, provides insight into how marker expression is spatially organized within a tissue. As an example, the notebooks illustrate calculating the keratin-keratin correlation for 10 nearest neighbors. In the manuscript, this corresponds to C_AB(r) with A=Keratin, B=Keratin, and r=10. For each cell, we calculate the mean Keratin value of the neighbors and append that value to our data frame. The resulting correlation is fairly high and is consistent with the results shown in the manuscript (manuscript Figure 2B). This supports the expected observation that cancer (Keratin-positive) cells tend to be spatially clustered near other cancer cells.

#### 3.4.3. Using GeoSpatial functions in BigQuery.

Along with the large number of BigQuery functions available for mathematical, statistical, and other applications, BigQuery also features a suite of geospatial analytics functions. Geospatial analysis has a long history and rich set of methods developed for spatial analysis of satellite, other earth imagery, socioeconomic, and environmental data captured in geospatial information systems (GIS). Given the similarity in structure between GIS data and cellular spatial data, both representing features with associated coordinates, we investigated how these geospatial tools could be used to analyze spatial cellular data.

In Notebook 16, we use BigQuery GIS functions to analyze melanoma samples using results from a study that utilized multiplexed whole-slide imaging analysis to characterize intermixed and graded morphological and molecular features in human melanoma cancer samples^18^.

In this example, we will focus on sample MEL1–1, as described in the manuscript, corresponding to **HTAN_Biospecimen_ID** *HTA7_1_3*. The relevant data can be found in the HMS level 4 melanoma mask table. This table contains estimated marker intensities extracted from cell segmentations, along with data on the centroid location coordinates (**X_centroid**, **Y_centroid**). These coordinates represent the location of cells in units of pixels, where 1 pixel corresponds to 0.65 μm. The multiplexed whole-slide slide image is 36,857 pixels in width and 25,808 pixels in height.

In this case, melanoma (tumor) cells can be distinguished by **SOX10, S100B**, and **CD63** protein abundance. To identify tumor cells, we apply predefined thresholds for the expression levels of these markers. We calibrated these thresholds manually and focused on a spatial region of *HTA7_1_3*, characterized as Invasive Melanoma (IM), delineated by the following corner coordinates: lower left (23076.9 px, 9615.3 px) and upper right (30384.6 px, 15000 px). (The region boundaries were obtained by visual matching and conversion of μm to px results in real-valued pixel coordinates).

BigQuery GIS functions that operate on point geometries require coordinates in terms of latitude and longitude. Therefore, points using units of pixels must be scaled to the geographical coordinate system (GCS). We set the lower left corner of the slide image at (0°, 0°) in the GCS, which is located in the Gulf of Guinea. The scaling is then defined by assigning the GCS coordinates to the upper right corner of the image, located at (36857 px, 25808 px).

As an initial attempt, we tested a simple scaling where 1000 pixels corresponded to 1° on Earth. This maps the upper right corner of the slide image at 25.808 °N 36.857 °E, near the Red Sea (Supplemental Figure 1). However, at this location, the resulting distortion in distances due to the curvature of the Earth is over 10%, resulting in the top of the slide image appearing 10% narrower than the bottom when using GIS-based distance calculations. To minimize distortion, we redefined the scaling so that the top of the slide image aligns with 0.1°N. This adjustment leads to a distortion of less than 1px over the entire image (Table 3, Appendix).

In the first step in the notebook, we scale centroid pixel coordinates by a factor of 1/258,080 to convert them to Earth angular degrees. These scaled coordinates are stored as a table of **ST_GeogPoint** objects, which define geospatial points in BigQuery. Points are labeled as *Tumor* or *Other* based on protein marker thresholds using a SQL query. To analyze cellular neighborhoods using BigQuery GIS functions, we set a radius of 20 μm in physical dimension, equating to 13.2 meters on the Earth. A query is performed that utilizes the **ST_DWithin** GIS function to generate a table in which rows correspond to pairs of cells that are closer than 13.2 meters.

It is important to highlight that HTA7_1_3 comprises over 1.1 million cells. The spatial neighborhood query described above yields a table with nearly 14 million cell pairings. Due to the size of this dataset, we avoid downloading the results and instead store the output in a new BigQuery table. Using BigQuery SQL, we utilize the table to compute, for each tumor cell, the number of neighboring cells that are also labeled as tumor. This allows us to determine the local tumor cell density and spatial organization. The distribution of nearest neighbors shows that most tumor cells in this Invasive Melanoma region are surrounded by approximately 10 other tumor cells.

**Figure 9.**
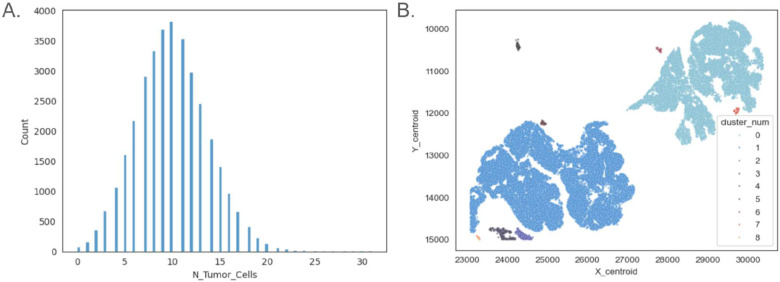
Examples **of melanoma tumor spatial analysis** (A.) Number of tumor cells within a 20 micrometer radius, for all tumor cells. (B.) Spatial cluster of tumor cells.

BigQuery’s geospatial analytics also provides additional functions such as ST_CLUSTERDBSCAN, which implements the DBSCAN algorithm to identify high-density spatial clusters. We use this function to identify spatial clusters of tumor cells. In Figure 9B, we display the resulting spatial clusters of tumor cells colored according to the cluster.

## 4. Discussion

The HTAN BigQuery resource brings together a large variety of data modalities into a single platform capable of massively parallel computation and integrative analysis, accommodating any budget and scale of question.

At the center of this resource is the HTAN metadata provenance table, which provides a central view into the multi-center data set and offers a structured approach to tracking and linking various data entities, and describes the history, source, and transformation of data objects. By consolidating information from numerous assay metadata tables into a single, queryable format, HTAN enables researchers to access comprehensive datasets

The HTAN ID provenance table serves as a powerful tool for navigating the HTAN data ecosystem. It not only connects clinical, biospecimen, and assay-related metadata tables, but also links data across multiple levels of processing, making it easier to track data lineage. The provenance table also facilitates reproducibility and traceability by linking sample identifiers across datasets to confirm the source and processing history of an assay or biospecimen. With this table, researchers can filter and extract specific information related to different assays, tissue samples, and processing levels, making cohort creation thorough and reproducible. With the high-level overview of HTAN data availability, it’s remarkably easy to count the number of available files for any given cohort, ensuring researchers have a clear understanding of the datasets before conducting deeper analyses.

Beyond assay metadata, the HTAN BigQuery resource also includes molecular data, such as single-cell sequencing. Rather than wide tables, which are often used in statistics software, data in BigQuery is transformed into “tidy formats”. In this structure, each row represents a single observation, which is typically a combination of sample, gene (or other feature), and its corresponding counts. Although these tables can be extremely long, BigQuery’s parallel processing engines allow for efficient calculations that can produce expression differences between cell or patient phenotypes.

By narrowing down a “cellular cohort”, users can extract and download data to create common analysis objects such as Scanpy AnnData objects compatible with packages such as CellTypist. When working with cloud-based computational notebooks, data transfer is fast and seamless, providing a smoother user experience. Additionally, the scATAC-seq data can be used for exploring cell state differences, such as comparing chromatin states between cell types and patient groups, without concerns about local disk capacity.

Spatial data can also be processed and analyzed in the BigQuery ecosystem, as evidenced by the GeoSpatial collection of built-in functions available. Currently, the HTAN BigQuery resource contains multiple spatial modalities with the location of cell segmentation masks and the marker intensity values extracted from those masks. This structure not only makes it possible to recreate published figures but also supports the development of spatial analyses and statistics using User Defined Functions (UDFs).

New data is released in the ISB-CGC resource following the HTAN release schedule. The current release is at version 6.2, but since the BigQuery data is versioned, analysis results can be reproduced from any previous data release, ensuring reproducibility. As data is released at HTAN, it is submitted to GC, the General Commons, which is part of the Cancer Research Data Commons (CRDC, https://dataservice.datacommons.cancer.gov). To query what data is present in the GC, the synapse id to DRS URI table can be used. This table contains a listing of all ~30,000 data files available in GC as of Release 4.0. Current notebooks are all for HTAN Phase 1, HTAN Phase 2 notebooks will be developed as data becomes available.

## Supplementary Material

1

## Figures and Tables

**Figure 3.**
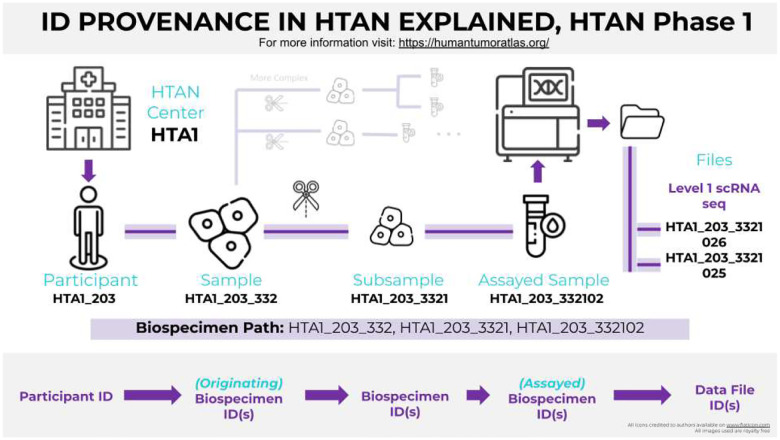
ID provenance in HTAN from participants to data files. The chain of provenance is captured in the HTAN ID provenance table in BigQuery, facilitating queries that relate stages of biospecimen extraction, processing and analysis.

**Figure 4.**
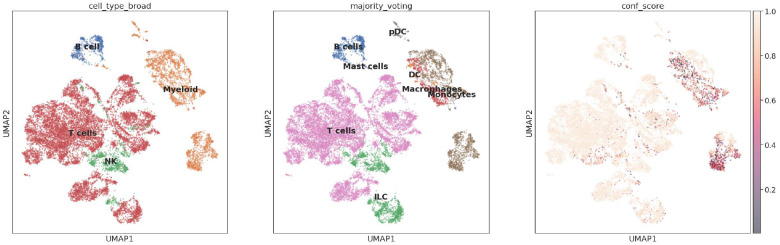
Predicting immune cell types with CellTypist and BigQuery Shown are single-cell UMAP plots with (A) Annotated cell types from the source publication, (B) predicted cell types, and (C) confidence scores for the predictions.

**Figure 5.**
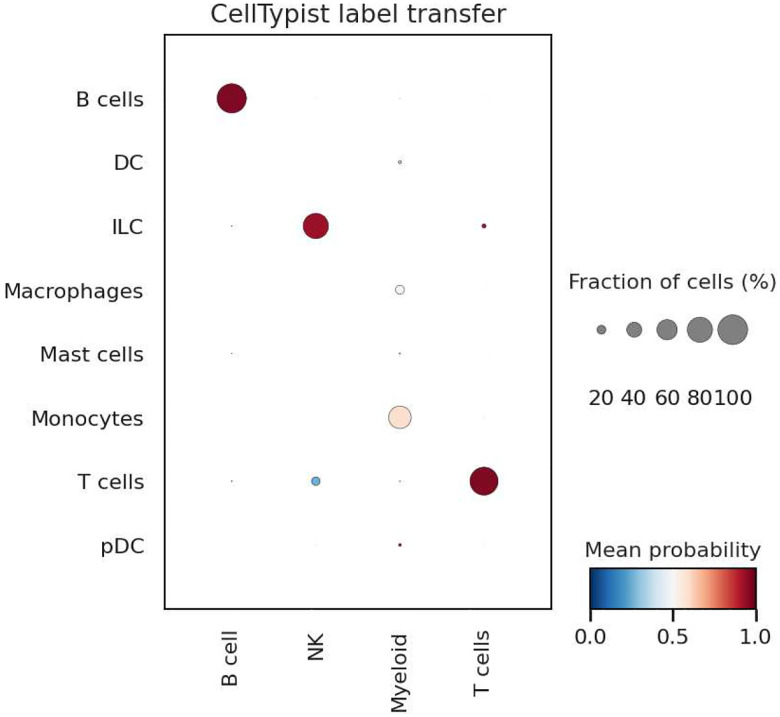
Comparing predicted and annotated immune cell types. A label transfer dotplot shows that the predicted labels are consistent with those provided from the generating center.

**Figure 6.**
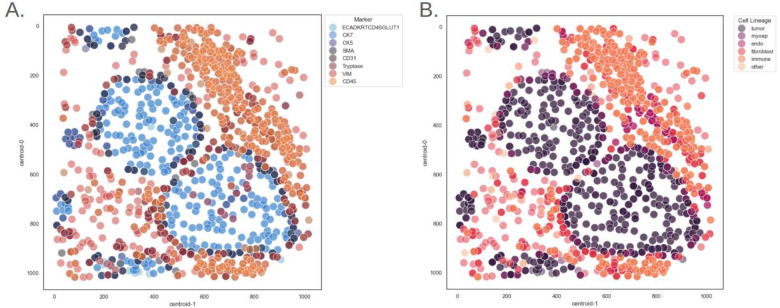
Plotting spatial cell phenotypes for breast ductal carcinoma in situ., (A) cells are colored by protein markers, (B) cells are colored by assigned cell types.

**Figure 7.**
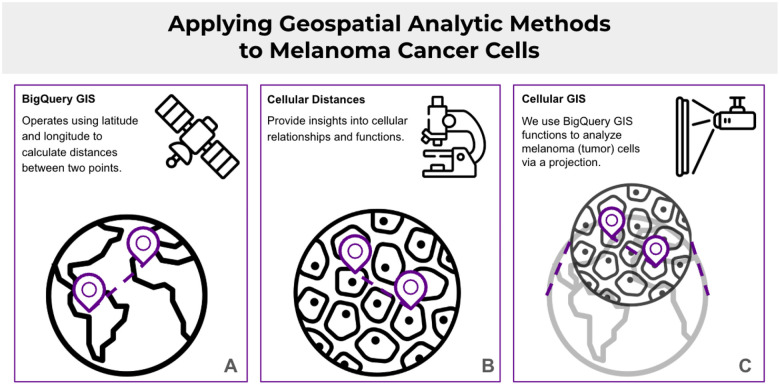
Application of geospatial functions in BigQuery to cell spatial data.

**Figure 8.**
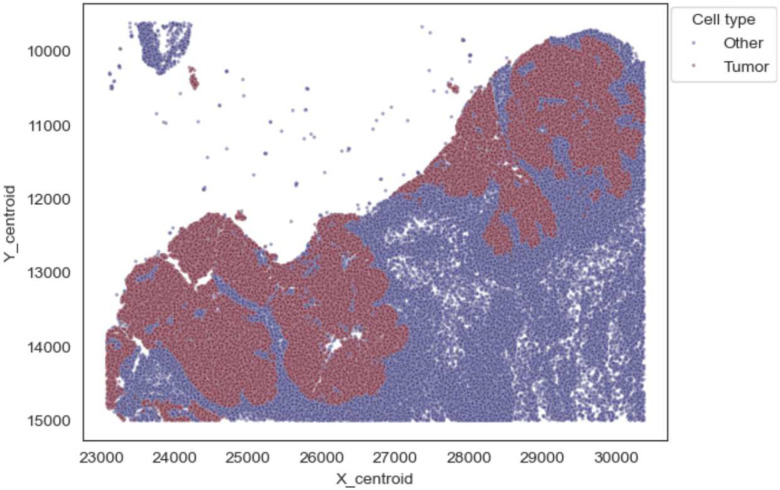
Melanoma Cell type clusters computed in BigQuery.

**Table 1.** List of available notebooks, ordered and grouped by section.

	Type	Notebook title
1	Getting started	ISB-CGC Quick Start Guide
2	Getting started	ISB-CGC BigQuery Tables
3	Getting started	A_Guide_to_HTAN_Data.ipynb
4	Provenance	HTAN_ID_Provenance_In_BQ.ipynb
5	Clinical	Explore_HTAN_Clinical_Biospecimen_Assay_Metadata.ipynb
6	By Organ	Identifying_HTAN_Data_Files_by_Organ_in_ISB_CGC.ipynb
7	Precancer	Identifying_and_Compiling_Precancer_Cases_and_Samples_in_HTAN.ipynb
8	Multi-modal	Cross_Modality_Metadata_Matching_in_HTAN_Datasets.ipynb
9	Data access	Creating_General_Commons_Data_Import_Manifests_Using_BQ.ipynb
10	Single Cell	Building_AnnData_with_Subset_of_Cells_from_BQ.ipynb
11	Single Cell	Investigating_Single_Cell_HTAN_Data.ipynb
12	Single Cell	Analyzing_HTAN_scRNASeq_Data_Using_CellTypist.ipynb
13	Single Cell	Analyzing_HTAN_scATACseq_Data_Using_SnapATAC2.ipynb
14	Spatial	Analyzing_HTAN_MIBI_Imaging_Data.ipynb
15	Spatial	Explore_HTAN_Spatial_Cellular_Relationships.Rmd
16	Spatial	Analyzing_HTAN_spatial_data_with_BigQuery_geospatial_analytics.ipynb

* Notebook numbers updated in manuscript

**Table 2.** Friendly table names mapping to BigQuery table addresses.

Manuscript mention	Description	BQ Friendly name	Table address
ID provenance	ID mapping table	HTAN ID PROVENANCE TABLE	isb-cgc-bq.HTAN.id_provenance_r6
synapse id to DRS URI	Map of synapse ID to DRS URIs	HTAN FILE SYNAPSE ID TO CDS DRS URI MAP	isb-cgc-bq.HTAN.cds_drs_map_r6
assay to BigQuery table map	Map of synapse IDs to BigQuery table IDs	HTAN ASSAY FILE SYNAPSE ID TO CGC BIGQUERY TABLE ID MAP	isb-cgc-bq.HTAN.dataFileSynapseID_to_BigQueryTableID_map_r6
tissue and organ mapping	Tissue and organ of origin across assays.	TISSUE OR ORGAN OF ORIGIN TO WHOLE ORGAN MAPPING TABLE	isb-cgc-bq.HTAN.tissueOrOrganOfOrigin_to_wholeOrgan_mapping_current
clinical tier 1 demographics	Patient demographics	HTAN CLINICAL TIER 1 DATA - DEMOGRAPHICS	isb-cgc-bq.HTAN.clinical_tier1_demographics_r6
tissue or organ of origin mapping	Tissue and organ systems nomenclature	TISSUE OR ORGAN OF ORIGIN TO WHOLE ORGAN MAPPING TABLE	isb-cgc-bq.HTAN.tissueOrOrganOfOrigin_to_wholeOrgan_mapping_r6
HMS level 4 melanoma mask	Cell level segmentations for melanoma tissue specimen.	HTAN HMS IMAGING LEVEL 4 DATA - MELANOMA WITH MASK	isb-cgc-bq.HTAN.imaging_level4_HMS_mel_mask_r6
imaging level 2	Metadata for Level 2 imaging data.	HTAN IMAGING LEVEL 2 METADATA	isb-cgc-bq.HTAN.imaging_level2_metadata_r6
clinical tier 1 diagnosis	Clinical table with patient diagnosis.	HTAN CLINICAL TIER 1 DATA - DIAGNOSIS	isb-cgc-bq.HTAN.clinical_tier1_diagnosis_r6
clinical tier 1 therapy	Clinical table with patient treatments.	HTAN CLINICAL TIER 1 DATA - THERAPY	isb-cgc-bq.HTAN.clinical_tier1_therapy_r6
scRNA-seq level 3	Single cell RNA-sequencing level 3 metadata.	HTAN SCRNASEQ LEVEL 3 METADATA	isb-cgc-bq.HTAN.scRNAseq_level3_metadata_r6
Duke level 4 mibi imaging	MIBI Imaging submitted by the Duke HTAN Center.	HTAN DUKE IMAGING LEVEL 4 DATA - MIBI	isb-cgc-bq.HTAN.imaging_level4_Duke_mibi_r6
HMS level 4 CRC mask	Cell level segmentations for colorectal cancer tissue specimen.	HTAN HMS IMAGING LEVEL 4 DATA - COLORECTAL WITH MASK	isb-cgc-bq.HTAN.imaging_level4_HMS_crc_mask_r6

**Table 3.** Number of tables and total number of cell transcriptomes captured in BigQuery.

HTAN Center	Number of Single Cell Sequencing Tables	Total Number of Rows	Total Number of Distinct Cells	Total Number of Rows
CHOP	2	1,023,803,936	52,671	1.02B
HTAPP	1	1,493,959	27,368	1.49M
MSK	11	910,608,000	439,957	910.61M
Vanderbilt	7	4,638,156,608	290,657	4.64B

**Table 4.** Linear segments on slide image and effects of mappings to the global coordinate system.

Linear Segment	GIS Mapping	Image dimension (Pixels)	Physical Dimension (*μ*m)	Earth Analog Dimension	Scale Factor	Distortion of 100,000 px for GIS Mapping
**Image Vertical**	Top of image at 0.1° N	25,808	16775.2	11.1 km	1.000001523	0.01523 px
**Image Vertical**	1000 px is 1° latitude	25,808	16775.2	2865 km	1.1107	1111 px
**Radius of Cell Neighborhood**	Top of image at 0.1° N	30.8	20	13.2 m	1.000001523	0.01523 px
**One pixel**	None	1	0.65	NA	NA	NA

## Data Availability

All data used in this journal are made publicly available through the HTAN Portal: https://humantumoratlas.org/, and the ISB-CGC: https://www.isb-cgc.org/ in the Google Public Dataset Project at isb-cgc-bq. BigQuery data referenced comes from data version 6.

## Code Availability

The associated Jupyter Notebooks and data tools provided are available via GitHub: https://github.com/ncihtan/Community-Notebooks/tree/v6.0.0

## Appendix - Distortions in Mapping slide image Coordinates to GCS coordinates

Distortion at latitude φ (in radians) is determined by the scale factor *k*. The scale factor *k* relates to the increase in size of depicted land masses away from the equator and towards the earth’s poles in 2D maps of the earth, as is familiar from the map of the world seen in grade school classrooms. The scale factor at a latitude φ (in radians) is *k* = 1/cos(φ) = sec(φ). In the commonly used Mercator projection (and in conformal mappings in general) the horizontal and vertical scale factors are identical. Opposite to the conventional mapping problem, in which a spheroid is mapped to a 2D surface, here we are mapping the (flat) slide image coordinates to the spheroid. However, the same considerations of scale distortion apply in this opposite direction.

Some details are provided here on the values shown in Table 4 and Notebook 16. Since we are only concerned with rough estimates of scale and distortion, we do not need to take into account the true shape of the earth (an irregularly shaped ellipsoid), can work in the spherical approximation, and with a limited number of significant digits in earth distance. The relation between angle and distance varies over the surface of the earth but for conversions we worked with the fixed value 1° = 111 km, consistent with what is measured for distance from equator to 1°N, to this significance. We “place” the bottom left corner of the slide image at the GCS origin (0°N 0°E, where the prime meridian and the equator intersect), which is south of Ghana, and in the Gulf of Guinea.

### Appendix 1. Slide image pixels and earth distance

Given an image width of 36,857px and a height of 25,808px.

1° N = 111 km

0.1° N = 11.1 km = 25,808 px

11.1 m = 25.808 px

1 m = 25.808/11.1 px = 2.325 px

1 px = 11.1/25.808 m = 0.4301 m

### Appendix 2. Distortion when top of slide image when mapped to 0.1° N

In radians φ = 0.1°

is φ = 2 π rad × 0.1°/360° rad = 0.001745 rad.

The Taylor expansion of *k*=sec(φ) around φ zero is

*k* = 1+ φ^2 / 2 + ….

For small angles, the distortion of 10,000 pixels is

φ^2 / 2 × 10,000 px = 0.01523 px.

The distortion of 25,808 pixels is

φ^2 / 2 × 25,808 px = 0.0393 px.

### Appendix 3. Neighborhood of 20 μm true physical radius

The true physical pixel length and width are 0.65 μm.

Then physically 20 μm = 20 μm / (0.65 μm/px) = 30.769 px.

The Earth equivalent

30.769 px * (0.4301 m/px) = 13.233 m (earth).

### Appendix 4. Distorted mapping example

Initially we mapped 1000 px to 1° on earth,

25,808px is 25.808° N

which gives φ = 25.808°/360° 2 π rad = 0.4504346 rad.

The scale factor at latitude 25.808°N is then

k = sec(φ) = 1.1107,

giving a distortion of 10,000px : 1,111px.

For context, this corresponds to the top right of the slide image being at 25.808 °N 36.857 °E (see Supplemental Figure 1). The red pin in Supp. Fig. 1 shows this location, with the angles in minutes and seconds (Google Earth).

## References

[1] Wang Z. NCI Cancer Research Data Commons: Resources to Share Key Cancer Data. Cancer Res. (2024) doi:10.1158/0008-5472.CAN-23-2468. PMC11063687 38488507

[2] Brady A. NCI Cancer Research Data Commons: Core Standards and Services. Cancer Res. 84, 1384–1387 (2024). 38488505 10.1158/0008-5472.CAN-23-2655 PMC11067691

[3] Pot D. NCI Cancer Research Data Commons: Cloud-Based Analytic Resources. Cancer Res. 84, 1396–1403 (2024). 38488504 10.1158/0008-5472.CAN-23-2657 PMC11063685

[4] Berisha B., Mëziu E. & Shabani I. Big data analytics in Cloud computing: an overview. J. Cloud Comput. Heidelb. Ger. 11, 24 (2022). 10.1186/s13677-022-00301-w PMC9362456 35966392

[5] Fernandes S. & Bernardino J. What is bigquery? in 202–203 (2015).

[6] Melnik S. Dremel: interactive analysis of web-scale datasets. Proc VLDB Endow 3, 330–339 (2010).

[7] de Bruijn I. Sharing data from the Human Tumor Atlas Network through standards, infrastructure and community engagement. Nat. Methods 22, 664–671 (2025). 40164800 10.1038/s41592-025-02643-0 PMC12125965

[8] Tomczak K., Czerwińska P. & Wiznerowicz M. The Cancer Genome Atlas (TCGA): an immeasurable source of knowledge. Contemp. Oncol. 19, A68 (2015). 10.5114/wo.2014.47136 PMC4322527 25691825

[9] Barnes C. Managing, Analyzing and Sharing Research Data with Gen3 Data Commons. Preprint at 10.48550/arXiv.2508.04944 (2025). PMC13454189 42156787

[10] Srivastava S., Wagner P. D., Hughes S. K. & Ghosh S. PreCancer Atlas: Present and Future. Cancer Prev. Res. (Phila. Pa.) 16, 379–384 (2023). 10.1158/1940-6207.CAPR-22-0435 37403657

[11] Wolf F. A., Angerer P. & Theis F. J. SCANPY: large-scale single-cell gene expression data analysis. Genome Biol. 19, 15 (2018). 29409532 10.1186/s13059-017-1382-0 PMC5802054

[12] Stuart T. Comprehensive Integration of Single-Cell Data. Cell 177, 1888–1902.e21 (2019). 31178118 10.1016/j.cell.2019.05.031 PMC6687398

[13] Domínguez Conde C. Cross-tissue immune cell analysis reveals tissue-specific features in humans. Science 376, eabl5197 (2022). 35549406 10.1126/science.abl5197 PMC7612735

[14] Chan J. M. Signatures of plasticity, metastasis, and immunosuppression in an atlas of human small cell lung cancer. Cancer Cell 39, 1479–1496.e18 (2021). 34653364 10.1016/j.ccell.2021.09.008 PMC8628860

[15] Morán-Plata F. J. Altered immune cell profiles in blood of mature/peripheral T-cell leukemia/lymphoma patients: an EuroFlow study. Front. Immunol. 16, 1561152 (2025). 40191194 10.3389/fimmu.2025.1561152 PMC11968749

[16] Risom T. Transition to invasive breast cancer is associated with progressive changes in the structure and composition of tumor stroma. Cell 185, 299–310.e18 (2022). 35063072 10.1016/j.cell.2021.12.023 PMC8792442

[17] Lin J.-R. Multiplexed 3D atlas of state transitions and immune interaction in colorectal cancer. Cell 186, 363–381.e19 (2023). 36669472 10.1016/j.cell.2022.12.028 PMC10019067

[18] Nirmal A. J. The Spatial Landscape of Progression and Immunoediting in Primary Melanoma at Single-Cell Resolution. Cancer Discov. 12, 1518–1541 (2022). 35404441 10.1158/2159-8290.CD-21-1357 PMC9167783

