# Supplementary material for: Analysis of clinical, single cell, and spatial data from the Human Tumor Atlas Network (HTAN) with massively distributed cloud-based queries
Source: Res Sq. 2025 Nov 17:rs.3.rs-7769205. Preprint. [Version 1] doi: 10.21203/rs.3.rs-7769205/v1 (PMC12668157; doi:10.21203/rs.3.rs-7769205/v1)
Supplement: 1 [file NIHPPRS7769205V1-supplement-1.pdf]

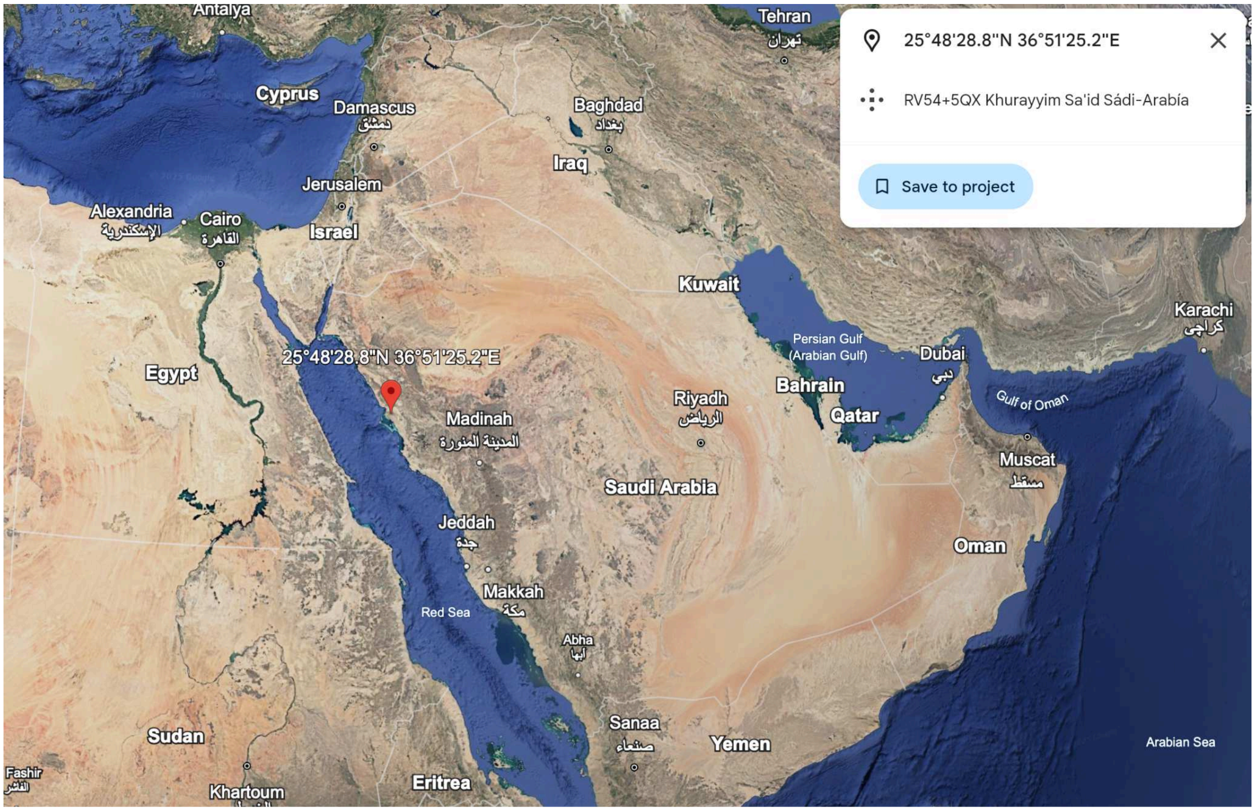

Supplemental Figure 1. **Earth location corresponding to mapping of image to GCS coordinates**. The red pin in the figure below shows where the top right corner of a reference image would be (Google Earth), if the bottom right corner is placed at the GCS origin and 1000 image pixels are mapped to 1º on earth This choice leads to an 11% distortion.
